# Millimeter-deep micron-resolution vibrational imaging by shortwave infrared photothermal microscopy

**DOI:** 10.21203/rs.3.rs-3449548/v1

**Published:** 2023-10-18

**Authors:** Ji-Xin Cheng, Hongli Ni, Yuhao Yuan, Mingsheng Li, Yifan Zhu, Xiaowei Ge, Jiaze Yin, Chinmayee Prabhu Dessai, Le Wang

**Affiliations:** Boston University; Boston Univeristy; Boston University; Boston University; Boston Univeristy; Boston University; Boston Univeristy; Boston University; Boston University

## Abstract

Deep-tissue chemical imaging plays a vital role in biological and medical applications. Here, we present a shortwave infrared photothermal (SWIP) microscope for millimeter-deep vibrational imaging with sub-micron lateral resolution and nanoparticle detection sensitivity. By pumping the overtone transition of carbon-hydrogen bonds and probing the subsequent photothermal lens with shortwave infrared light, SWIP can obtain chemical contrast from polymer particles located millimeter-deep in a highly scattering phantom. By fast digitization of the optically probed signal, the amplitude of the photothermal signal is shown to be 63 times larger than that of the photoacoustic signal, thus enabling highly sensitive detection of nanoscale objects. SWIP can resolve the intracellular lipids across an intact tumor spheroid and the layered structure in millimeter-thick liver, skin, brain, and breast tissues. Together, SWIP microscopy fills a gap in vibrational imaging with sub-cellular resolution and millimeter-level penetration, which heralds broad potential for life science and clinical applications.

## Introduction

Probing cellular activities and functions in intact tissue environments is crucial for a variety of biomedical applications such as cancer pathology and drug discovery ^1^. Vibrational microscopy is a powerful tool for studying cellular functions by providing chemical contrast from nutrients, metabolites, and other biomolecules ^2^. However, the imaging depth of current vibrational microscopy is not sufficient to map the chemical content in an intact organoid or a tissue without altering the natural microenvironment. Specifically, infrared spectroscopy-based approaches suffer from a strong water absorption which restricts the penetration depth to tens of micrometers ^3^. Spontaneous or coherent Raman microscopy based on visible or near-infrared excitation circumvents the water absorption issue. Nevertheless, the large tissue scattering limits their imaging depth to around 100 μm ^4, 5^. With spatially offset detection of diffusively back-scattered photons, spatially offset Raman spectroscopy ^6, 7^ and spontaneous Raman tomography ^8, 9, 10^ can acquire signals beyond millimeter-deep in a tissue. However, these methods only offer a millimeter-level spatial resolution, not sufficient to monitor cellular-level activity. In addition, their sensitivity is limited by the extremely small spontaneous Raman scattering cross-section.

**Figure 1:**
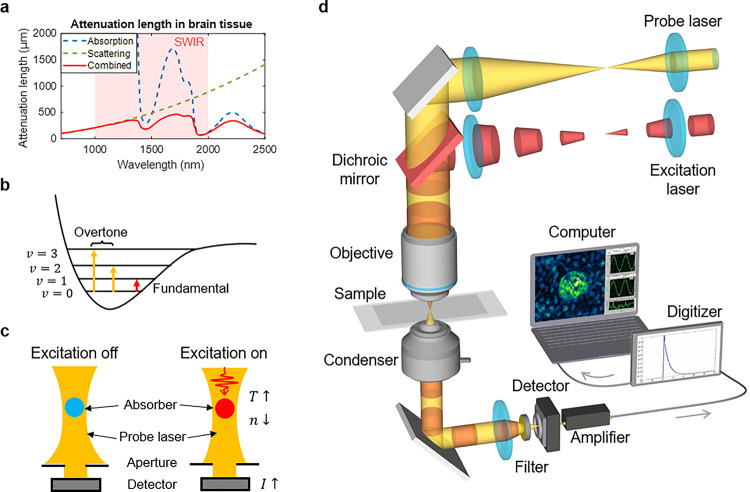
SWIP microscope principle and schematic. (a) Wavelength-dependent attenuation length in brain tissue calculated with water absorption and brain tissue scattering coefficients ^12^. (b) Overtone absorption energy diagram. (c) Photothermal contrast mechanism. T: temperature, n: refractive index, I: light intensity. (d) Short-wave infrared photothermal microscope schematic.

The shortwave infrared (SWIR) region (from 1000 to 2000 nm) ^11^ opens a new window for deep tissue imaging with much reduced scattering compared to the visible region and much reduced water absorption compared to the mid-infrared region (Fig. 1a) ^12, 13^. Importantly, the overtone transitions, which are high-order harmonics of the fundamental modes of molecular vibrations, of carbon-hydrogen (C-H) stretching (Fig. 1b) reside in this window ^14^, allowing deep vibrational imaging. Among various SWIR modalities, diffuse optical tomography can acquire images beyond millimeter-deep in tissue, yet at millimeter-level spatial resolution ^15^. Photoacoustic (PA) imaging achieves a higher spatial resolution by detecting acoustic waves with low tissue scattering ^16, 17^. SWIR-photoacoustic microscopy (PAM) allowed vibrational mapping of lipids in arterial tissues and drosophila embryo ^13^. Nevertheless, its sensitivity is not sufficient for visualizing subcellular features. In SWIR-PAM, the transducer is placed at a considerable distance away from the absorption site. A dramatic acoustic signal loss takes place during the propagation, which eventually degrades the detection sensitivity and constrains the ability to detect targets smaller than tens of micrometers. Additionally, the requirement of acoustic coupling mediums between the sample and transducer complicates the optical path design and is not applicable to a sample sensitive to mechanical contact such as a patient wound ^18^. Optically probed photoacoustic spectroscopy is developed for remote sensing purpose ^18, 19^ and has been extended to the SWIR window ^20, 21^. However, the sensitivity of photoacoustic remote sensing is not sufficient for subcellular chemical imaging.

Here, we present a shortwave infrared photothermal (SWIP) microscope that offers subcellular resolution, nanoparticle detection sensitivity, and millimeter-deep tissue imaging capability. By optically sensing the refractive index (RI) change directly from the absorption site (Fig. 1c), SWIP prevents signal loss during propagation and eliminates the necessity of sample contact. SWIP utilizes tightly focused SWIR lasers to achieve subcellular spatial resolution and millimeter-level imaging depth in highly scattering mediums, which allows imaging of single 1-μm polystyrene (PS) beads through 800-μm thick scattering phantom. Through high-speed signal digitization, we found the optically probed photothermal (PT) signal is 63 times greater in amplitude than the optically probed PA signal, which enables the nanoparticle detection sensitivity of SWIP. Furthermore, the PT dynamics detection in SWIP achieves the extraction of signals from small objects over surrounding medium background. With these advances, we demonstrate volumetric SWIP imaging of intracellular lipids across an intact tumor spheroid, lipids in thick animal tissue slices, and multilayer of fat cells in human breast biopsy.

## Results

### A shortwave infrared photothermal microscope

To fulfill the SWIP concept, we built a pump-probe microscope as shown in Fig. 1d. A 1725 nm laser with 10 ns pulse duration and 2 kHz repetition rate serves as the excitation laser to target the first overtone absorption of C-H vibration. A 1310 nm continuous wave (CW) laser is used as the probe. Although the first C-H overtone absorption cross section is around two orders of magnitude smaller than the fundamental absorption ^22, 23^, detecting at the first overtone region can circumvent the strong water absorption in the mid-infrared region where water absorption is more than 3 orders stronger than that in the SWIR region ^24^. Moreover, compared to the second overtone, the first overtone of C-H gives 7 times larger signal from lipids ^25^.

The excitation and probe beams are combined and focused into the sample to obtain the SWIP signal. The SWIP contrast originates from the absorption-induced thermo-optic effect. The thermal-modulated RI forms a micro-lens and consequently alters the propagation of the probe laser, which is eventually turned into a light intensity modulation by collecting light through a small aperture inside the condenser (Fig. 1d).

### Optically detected photothermal versus photoacoustic signal

Under pulsed excitation, PT and PA conversions occur simultaneously. Studying the relationship between the coupled PT and PA signals is valuable in designing the detection scheme. Yet, it is challenging to compare PT signal with transducer-detected PA signal due to the limited detector bandwidth and the complexity of a multi-modal system. Instead, optical detection and high-speed digitization offer an unprecedented bandwidth and can simultaneously monitor the PT and PA signals generated by thermo-optic or elasto-optic effects, respectively.

We first performed a theoretical analysis of the PT and PA contribution to the optically probed signal. Since both PT and PA signal can be written as a function of temperature rise, an amplitude ratio between optically probed PT and PA signal intensities could be calculated (Detailed in Supplemental Material):

#(1) $$\frac{I_{PT}}{I_{PA}}=\left|\frac{\delta n_{PT}}{\delta n_{PA}}\right|=\frac{2|\alpha |v_{a}^{3}\tau_{pulse}}{\eta n_{0}^{3}\Gamma C_{V}r_{focus}}$$

Here, α is thermo-optic coefficient, $v_{a}$ is speed of sound, $\tau_{pulse}$ is the pulse duration, η is elasto-optic coefficient, $n_{0}$ is the initial RI of the ample, Γ is Gruneisen parameter, $C_{V}$ is constant volume heat capacity, $r_{\mathrm{focus}}$ is the radius of probe focus. For olive oil, $|\alpha |=0.00043K^{-1}$,$\;v_{a}=1490m/s,\;\eta \approx 0.3$,$\;n_{0}=1.42,\;\Gamma =0.9,\;C_{V}=1970J*K*{\;kg}^{-1}$. Assuming $r_{\mathrm{focus}}=526nm$ calculated from numerical aperture of the objective, we find that $\frac{I_{PT}}{I_{PA}}\approx 37$.

**Figure 2:**
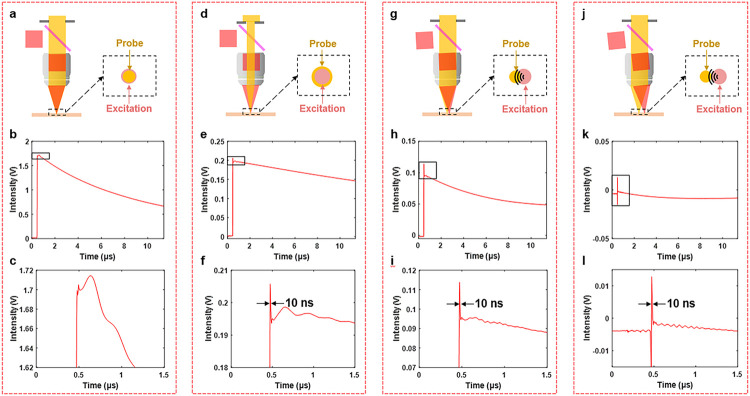
Comparison of optically detected photothermal and photoacoustic signals. (a) Normal SWIP focusing configuration where probe and excitation foci have similar size and good lateral overlapping. (b) Signal trace under the configuration in (a). (c) Zoom-in of (b). (d) Focusing configuration where the probe focus is enlarged. (e) Signal trace under the configuration in (d). (f) Zoom-in of (e). (g) Focusing configuration where probe focus has a small lateral shift relative to the excitation focus. (h) Signal trace under the configuration in (g). (i) Zoom-in of (h). (j) Focusing configuration where probe focus has a large lateral shift to the excitation focus. (k) Signal trace under the configuration in (j). (l) Zoom-in of (k). Sample: Olive oil. SWIR excitation power on sample: 4.2 mW.

The theoretical analysis indicates the PT contribution is more than one order larger than the PA contribution. To validate this result, we measured the PA and PT signals simultaneously using the SWIP microscope shown in Fig. 1d. Through fast digitization, the PT and PA contributions can be differentiated by their temporal profiles (Fig. 2). Because the estimated acoustic relaxation time is shorter than the pulse duration, the PA initial pressure rise should have the same duration as the excitation pulse, which is 10 ns. In contrast, the PT signal exhibits a long exponential decay as indicated by Newton’s law of cooling. When the two foci were tight and in a good lateral overlapping (Fig. 2a), the PT signal was found to overwhelm the PA signal (Fig. 2b). When zooming in the bounding box area of Fig. 2b, a bipolar PA oscillation was observed, but with an amplitude that is 63 times smaller than that of PT (Fig. 2c).

In order to confirm that the initial oscillation signal was indeed from PA, we acquired 3 other signal traces under different focusing conditions. We consider PT as a locally confined signal and PA as a propagating signal according to their diffusion speeds. Therefore, enlarging the probe focus size (Fig. 2d) or shifting the probe focus laterally out of the excitation focus (Fig. 2g and 2j) should selectively probe the PA signal. As expected, with the modified schemes, the relative amplitude of PA became larger in the detected signal (Fig. 2e, 2h, 2k), which could be clearly observed in the zoom-in views (Fig. 2f, 2i, 2l). In an extreme case, when the probe focus had zero overlap with the excitation focus, the PT contribution in the probed signal was negligible and a typical acoustic bipolar oscillation was observed (Fig. 2j–l). This result confirms the PA origin of the probed signal shown in Fig. 2l. The width of the strongest oscillation band in Fig. 2f, 2i, 2l was measured to be around 10 ns, consistent with the initial pressure rise theory. A ball-lens model interpreting the SWIP contrast under various focusing conditions can be found in Supplementary Material.

We noticed a 2 times mismatch between the theoretical calculation and the experimental result. This discrepancy is possibly due to the deviation of the elasto-optic coefficient used in our calculation from the real value in our experimental system. Another possibility is the limited bandwidth of our photodetector (70 MHz). Nevertheless, this mismatch does not influence the conclusion that the PT signal has more than one order larger amplitude than the PA signal in a normal focusing configuration shown in Fig. 2a.

### SWIP imaging characteristics

**Figure 3:**
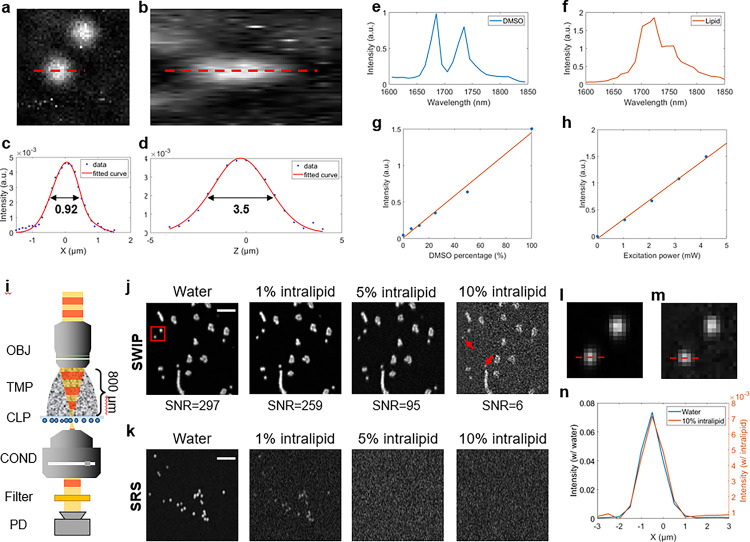
SWIP microscope performance. (a, b) XY and YZ section of volumetric SWIP image of single 500 nm PS beads. Excitation power on sample: 20 mW. (c, d) Single 500 nm PS bead’s lateral and axial profile corresponding to dashed lines in (a) (b). (e, f) SWIP spectra of pure DMSO and glycerol trioleate. (g) SWIP signal dependence on concentration. Sample: DMSO solution in DMSO-D6. (h) SWIP signal dependence on excitation power. Sample: pure DMSO. Excitation power on sample: 4.2 mW. (i) Scattering phantom imaging schematic. OBJ: objective lens. TMP: tissue-mimicking phantom. CLP: Coverslip. COND: condenser. PD: photodiode. (j) SWIP imaging results of 1.0 μm PS beads through water or scattering medium. Laser power on sample: 1725 nm: 20 mW. 1310 nm: 10.3 mW. (k) SRS imaging results of 1.0 μm PS beads through water or scattering medium. Laser power on sample: 798 nm: 10 mW. 1040 nm: 60 mW (water group); 798 nm: 50 mW. 1040 nm: 100 mW (intralipid groups). Scale bar: 10 μm. (l) Zoom-in view of the red box in (j). (m) SWIP imaging result through 10% intralipid solution in the same field-of-view of (l). The probe power is increased to get better SNR. 1725 nm: 20 mW. 1310 nm: 97.4 mW. (n) Lateral profile of single 1.0 μm PS bead as indicated by the red dashed lines in (l) and (m). Blue line is corresponding to (l). Orange line is corresponding to (m).

We have characterized the performance of our SWIP microscope using standard samples. Figure 3a and 3b show the XY section and YZ section of volumetric SWIP imaging of single 500 nm PS beads. The signal-to-noise ratio (SNR) of the XY image of a bead was 25, which shows a high sensitivity of SWIP in detecting nano-objects. Figure 3c and 3d show the lateral and axial profile of a single 500 nm PS bead, where the lateral and axial FWHM were measured to be 0.92 μm and 3.5 μm. After deconvolution with the beads profile, the system’s lateral and axial resolution were calculated to be 0.77 μm and 3.5 μm. Such a resolution is sufficient to resolve the subcellular features.

To confirm that the SWIP contrast is indeed from C-H overtone absorption, we acquired SWIP spectra of standard samples (Fig. 3e and 3f ), which match the literature well ^11, 26^. Figure 3g and 3h show a linear dependence of the SWIP signal on both the excitation power and the molecular concentration, in consistence with the ball-lens model of SWIP contrast (Supplementary Material). Collectively, Fig. 3e–h demonstrate the quantitative chemical analysis capability of SWIP microscopy.

The deep-penetration, high-resolution imaging capability of SWIP was characterized with scattering phantoms. A tissue-mimicking intralipid aqueous solution was placed between the objective and 1.0 μm PS beads dried on a coverslip (Fig. 3i). According to literature, 1% intralipid has a similar scattering coefficient to human skin epidermis ^27^. Figure 3j shows the SWIP imaging results through water, 1% intralipid, 5% intralipid or 10% intralipid. SWIP successfully resolved single 1.0 μm PS beads even under 10% intralipids as pointed by the red arrow. For comparison, near-infrared stimulated Raman scattering (SRS) imaging was also performed under the same condition (Fig. 3k). In pure water condition, the SRS image showed higher resolution than the SWIP image due to shorter excitation wavelengths. However, the quality of SRS images quickly degraded as the intralipid concentration increased. No beads could be seen by SRS under 5% and 10% intralipid solutions. To investigate the resolution degradation of SWIP through scattering medium, we took a zoom-in view at the red box in Fig. 3j for single 1.0 μm PS SWIP image through water (Fig. 3l). Figure 3m shows the same field-of-view through 10% intralipid, in which the probe power was increased compared to Fig. 3j to obtain better SNR for resolution characterization. Figure 3n shows a comparison between single bead lateral profile in Fig. 3l and Fig. 3m, where the bead profile shows no significant broadening through 10% intralipid, indicating the SWIP maintained good spatial resolution through highly-scattered medium. Together, Fig. 3j–n show that SWIP has deep-penetration, high-resolution imaging capability through a highly scattering medium, which is beyond reach of a commonly used SRS microscope.

### SWIP imaging of intracellular lipids in an intact tumor spheroid

Tumor-derived spheroid is a kind of in-vitro cancer model that better recapitulates tumor physiology and response than two-dimensional cell culture ^28^. As cancer development is closely related to the altered lipid metabolism ^29^, imaging intracellular lipids inside a spheroid can help understand cancer progression and test drug effectiveness. However, imaging cellular components inside a spheroid is challenging as the densely packed cells strongly scatter the light. Sectioning ^30^ and tissue clearing ^31, 32^ have been applied to circumvent the strong scattering. Yet, the sectioning and clearing methods may alter the metabolic state of the spheroid and cannot be used for live sample study. Deep-penetrating multi-photon fluorescence microscopy and light-sheet fluorescence microscopy can image live spheroids ^33, 34^. However, the fluorescent labeling is perturbative, especially for small lipid molecules ^35^.

**Figure 4:**
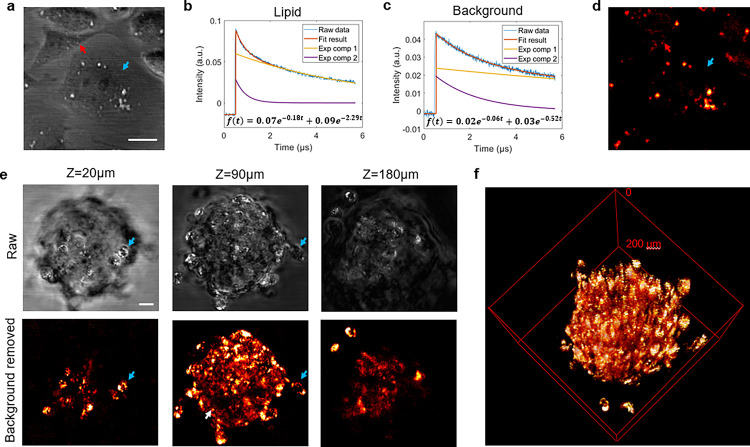
SWIP imaging of cancer cells and spheroid. (a) Raw SWIP image of monolayer OVCAR-5-cisR cells. Red arrow: lipid droplets. Blue arrow: water area. (b) Single-pixel SWIP signal trace at the red arrow-pointed lipid area. (c) Single-pixel SWIP signal trace at the blue arrow-pointed background area. (d) Background rejection result of (a) using the decay characteristic. Red arrow: lipid droplets. Blue arrow: water area. (e) SWIP imaging of an OVCAR-5-cisR spheroid. (f) 3-D rendering of volumetric SWIP imaging of the OVCAR-5-cisR spheroid after background rejection. Laser power on sample: 1725 nm: 20 mW, 1310 nm: 8.5 mW. Scale bar: 20 μm.

SWIP provides an opportunity to overcome the above-mentioned challenges by its micron-resolution, millimeter-deep vibrational imaging capability. Before imaging the spheroid, we first validated the SWIP contrast on single-layer cell culture. As shown in Fig. 4a, SWIP well mapped intracellular lipids and revealed good cell morphology. Utilizing the different thermal decay coefficients of lipids (Fig. 4b) and bulk water (Fig. 4c), the water background in Fig. 4a can be removed (blue arrow, Fig. 4d) and the intracellular lipid contrast is much enhanced (red arrow, Fig. 4d). The logic and workflow of the background suppression can be found in the Methods.

Figure 4e shows SWIP imaging result of an intact tumor spheroid at 3 representative depths. In the raw SWIP images, the intracellular lipids were successfully identified at three depths representing the top, equatorial, and bottom planes. After removing the water background, the intracellular lipids showed up cleanly. Individual cells can be identified by circles of intracellular lipids (blue arrow in Fig. 4e). Some hollow structure in the center of the spheroid can also be observed (white arrow in Fig. 4e). Figure 4f is a background-removed three-dimensional rendering for the volumetric SWIP images of the spheroid. The volumetric SWIP image shows an enriched accumulation and a relative uniform distribution of lipid across the spheroid.

### SWIP imaging of lipids in biological tissues

Lipids play an important role in biological tissue including energy storage, signaling, and transport of fat-soluble nutrients ^36^. Imaging lipid content and its distribution inside a tissue can enable a range of applications ^37^. Because fluorescence labeling is perturbative for the lipid molecules, vibrational imaging is widely adopted for lipid studies ^35^. As reviewed in the introduction, current vibrational imaging modalities do not allow high-resolution lipid imaging in deep tissue. Consequently, tissue sectioning is generally applied to allow high-resolution layer-by-layer imaging, but the sectioning process usually introduces morphological artifacts and often causes lipid loss ^38^.

**Figure 5:**
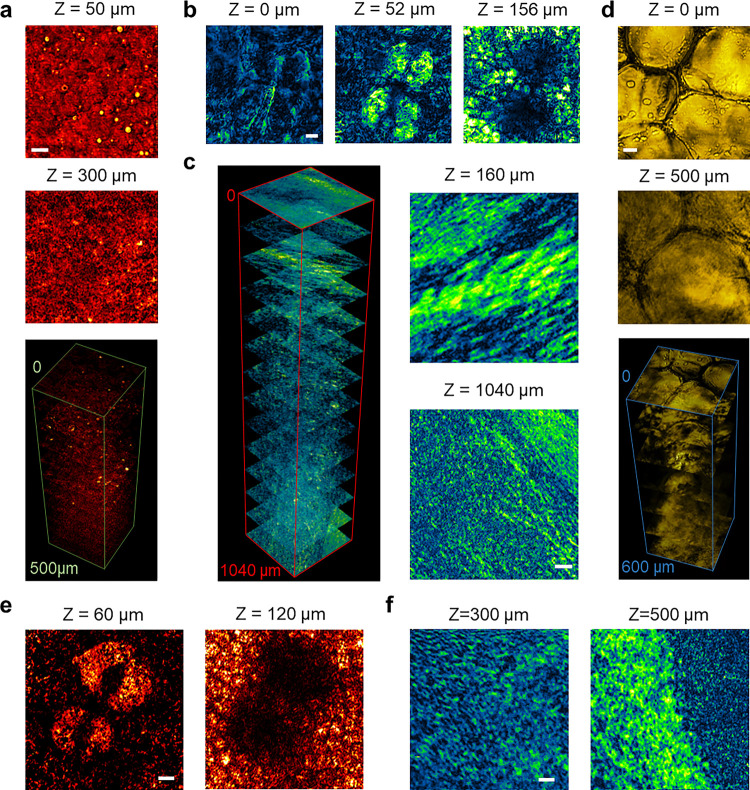
SWIP imaging of in biological tissues. (a) SWIP imaging of a fresh swine liver slice. (b) SWIP imaging of a mouse ear. (c) SWIP imaging of a mouse brain slice. (d) SWIP imaging of a breast biopsy from a healthy human. (e) Epi-SWIP imaging of a mouse ear at 60 μm and 120 μm depth. (f) Epi-SWIP imaging of a mouse brain at 300 μm and 500 μm depth. Laser power on sample: 1725 nm: 20 mW, 1310 nm: 70 mW. Scale bar: 20 μm.

To overcome the above-mentioned challenges, we explored SWIP imaging of lipids in various types of tissues. Figure 5a shows SWIP images of a fresh swine liver slice. The lipid and liver morphology revealed by SWIP was consistent with previously reported SRS results ^39^. Lipid droplets as small as 1.0 μm in diameter can be distinguished even at 300 μm deep inside the fresh liver with rich blood content, which cannot be achieved via existing modalities. Figure 5b shows SWIP images of a mouse ear. Characteristic layered structures were observed, including hair at Z=0μm, sebaceous gland at Z=52μm, and subcutaneous fat layer or cartilage at Z=156μm. Figure 5c reports the imaging result on a mouse brain slice with a thickness of around 1.0 mm. SWIP can image through the whole brain slice and well capture the myelin fibrous structure. Figure 5d demonstrates SWIP imaging on a breast biopsy of a healthy human. Different layers of fat cells can be resolved across a 600 μm thick breast tissue.

Finally, towards in vivo deep-tissue imaging, we have built and tested an epi-detected SWIP system with its schematic shown in **Fig. S3**. A major challenge facing epi-detection is the weak back-scattered probe laser intensity, which gives a poor SNR when a normal biased photodiode is used. To address this issue, we harnessed an amplified photodiode with higher sensitivity and achieved high-performance epi-detection. Figure 5e shows epi-detected SWIP images of a mouse ear at the depth of 60 μm and 120 μm. The epi-imaged sebaceous gland at Z=60μm and subcutaneous fat layer or cartilage at Z=120μm show a good match with the forward result (Fig. 5b). Figure 5f reports epi-detected SWIP images of an intact mouse brain. Cell-like features were observed at the depth of 300 μm. The bright myelin-like features appeared at the depth of 500 μm, where the imaging field of view captured the transition from the cell-rich grey matter to the lipid-rich white matter. Together, these results show that in-vivo SWIP imaging could be potentially achieved.

## Discussion

**Figure 6:**
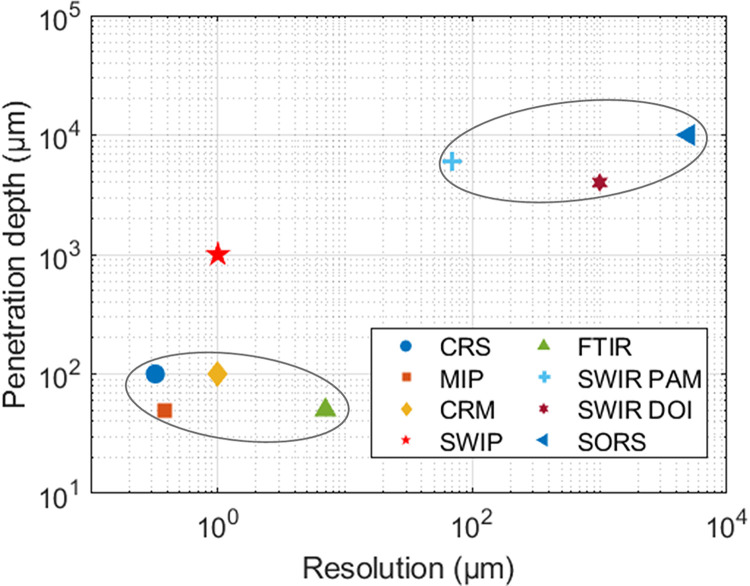
Penetration depth versus spatial resolution of vibrational imaging modalities. The data of penetration depth and spatial resolution are collected from reference ^3, 4, 6, 15, 16, 40^. CRS: coherent Raman scattering, MIP: mid-infrared photothermal, CRM: confocal Raman microscopy, FTIR: Fourier transform infrared spectroscopy, SWIR PAM: short-wave infrared photoacoustic microscopy, SWIR DOI: shortwave infrared diffusive optical imaging, SORS: spatial-offset Raman spectroscopy, SWIP: shortwave infrared photothermal.

A comparison of SWIP with existing vibrational imaging modalities in the dimension of penetration depth versus spatial resolution is illustrated in Fig. 6. These techniques can be clustered into three groups. The first group in the bottom-left has subcellular resolution and sensitivity but limited imaging depth on the scale of tens of microns to 100 microns. The second group in the top-right has deep penetration depth but relatively poor spatial resolution on the scale of hundreds of microns to several millimeters. Although SWIR PAM can achieve higher spatial resolution with a tight optical focus, the large signal loss eventually prevents SWIR PAM from probing small intracellular components. Clearly, there exists a gap between the first group and the second group for deep tissue vibrational imaging at subcellular resolution. As demonstrated in this work, SWIP successfully fills in the gap between the first and second groups. The millimeter-deep, micron-resolution, high-sensitivity vibrational imaging capability provided by SWIP opens exciting opportunities for many applications including live organoid study, slice-free tissue pathology, dynamic embryo imaging, etc.

It is noteworthy that the SWIP signal is sensitive to the focusing condition. As shown in Fig. 2, the best configuration for PT detection is when the two foci have similar size and in good lateral overlapping. The PA signal can be selectively detected if the probe volume is larger than the excitation volume. This is the case for the photoacoustic remote sensing study ^18^ where the excitation and probe wavelengths were 532 nm and 1310 nm, respectively. Compared to PT, PA imaging has its own merits. The PA signal is closely related to the mechanical property which is valuable for many applications ^41^. Furthermore, the high-frequency characteristics of PA can circumvent low-frequency noises. Our data and theory relating the signal level with the focusing condition can guide future system design for better utilization of the different strengths of PT and PA processes.

The reported SWIP microscope can be improved in several aspects. By far, our single-color SWIP imaging using the first overtone vibration at 1725 nm mainly targets the C-H bond that is enriched in lipid content. With a wavelength-tunable excitation laser, hyperspectral SWIP imaging and subsequent decomposition can differentiate multiple molecular species such as proteins, fatty acids, cholesterol, and carbohydrates ^11, 14^. Second, the acquisition time of current SWIP microscope is around 3 minutes per frame, limited by the 2 kHz excitation laser repetition rate. Combining a higher-repetition-rate excitation laser with galvo scanning, the imaging speed of SWIP can be much improved. Third, SWIP has shown good performance in imaging through a homogeneous scattering phantom but encounters image degradation at hundreds-of-micrometers deep in the tissue. We attribute such degradation to tissue-induced aberration which inevitably distorts the laser focus. By implementing adaptive optics for aberration correction ^42^, SWIP can reach even deeper into the tissue. Lastly, we envision that SWIP microscopy can be upgraded into SWIP-OCT for high-speed volumetric vibrational imaging of organoids and tissues.

## Methods

### Chemicals

The oil sample was prepared by sandwiching 2 μl vegetable oil with two no.1 coverslips. An 80 μm double sided tape was placed between the two coverslips as a spacer. To prepare the polystyrene (PS) beads sample, an aqueous PS beads solution was first prepared and mixed well with an ultrasound homogenizer to avoid large aggregates. Then the beads solution was dried on a no.1 coverslip. When performing SWIP imaging, the coverslip side was on the top to isolate the beads from the immersion medium or the scattering phantom.

### Cancer cells and spheroids

Cisplatin-resistant ovarian cancer cells (OVCAR-5-cisR) ^43^ were generously provided by the Daniela Matei Lab at Northwestern University. The cells were cultured in RPMI 1640 with 10% fetal bovine serum, 100 units/ml penicillin and 100 μg/ml streptomycin. Cells were seeded on a coverslip for 24 hours for monolayer cell imaging. To form ovarian cancer spheroids, 200 μl/well of OVCAR-5-cisR cell suspension were added to an ultra-low attachment 96 well plate with a cell density of 0.5 × 10^4^ cells/ml. The spheroids were cultured for 7 days. Cells were then kept in 1× Phosphate Buffered Saline (PBS) and sealed in between two coverslips with spacers.

### Biological tissues

The fresh swine liver was purchased from the supermarket. Before imaging, the liver was hand-sliced into around 3 mm and sandwiched between two no.1 coverslips. The mouse ear sample was isolated from a 6-month-old mouse and fixed with 10% formalin solution. Before imaging, the ear was rinsed with 1× PBS and then attached to a coverslip. The mouse brain was from a 6-month-old mouse and fixed with 10% formalin solution. The brain was sliced to be 1-mm thick with a vibratome. Before imaging, the brain was rinsed with 1× PBS then attached to a coverslip. The de-identified healthy human breast biopsy sample was obtained from Susan G. Komen tissue Bank at the IU Simon Cancer Center. The breast biopsy sample was freshly frozen and had a thickness of around ~ 0.6 mm. Before imaging, the breast biopsy sample was defrosted then sandwiched between two coverslips.

### SWIP microscope

Both pump and probe beams are in the shortwave infrared window to ensure deep tissue penetration. The pulsed pump beam is generated by an optical parametric oscillator (DX-1725-OPO, Photonics Industries), with a repetition rate of 2 kHz, wavelength centered at 1725 nm, and a pulse duration of 10 ns. The 1310 nm probe beam is provided by a CW diode laser (TURN-KEY CCS-LN/1310LD-4-0-0/OC, Research Lab Source Corporation). The 1725 nm excitation laser is chosen to excite the first overtone of C-H stretching vibrations.

The beam sizes of the two lasers were adjusted to be around 6 mm with telescopes. The telescopes are also used for optimizing the axial offset of the two laser foci on sample to obtain maximum SWIP signal ^44^. After beam expansion, the two lasers were collinearly combined with a dichroic mirror and then focused into a sample through an objective lens. Two objective lenses were used for the experiments. A 1.0 NA water-immersion objective with 800 μm working distance was used to image the polymer beads, cancer cells, spheroid, swine liver, and human breast biopsy. A 2-mm working distance objective with an effective NA of ~ 0.4 was applied for mouse ear and brain imaging. The transmitted light from the sample was collected by an air condenser (D-CUO, Nikon) with an adjustable aperture. After the condenser, the remaining excitation laser was filtered out by a 1310 nm bandpass filter (FBH1310–12, Thorlabs). The signal-carrying probe beam is detected by a biased InGaAs photodiode (PD). When recording both PA and PT signals, we used a small-area high-speed PD (70 MHz bandwidth, 0.8 mm^2^, DET10N2, Thorlabs). When only targeting the PT signal, we used a slower PD with larger active area (11.7 MHz bandwidth, 3.14 mm^2^, DET20C2, Thorlabs). The photocurrent from the PD is converted to a voltage signal with a 50 Ohm impedance and then amplified by an AC-coupled low-noise voltage amplifier (100 MHz bandwidth, SA230F5, NF corporation) and digitized by a high-speed data acquisition card at 180 MSa/s (ATS9462, Alazar Tech). Every excitation laser pulse corresponds to a single pixel in the image. The image was formed by sample scanning achieved with a stage (Nano-Bio 2200, Mad City Labs). The volumetric image was acquired with a motorized z-knob to allow axial scanning.

### SWIP image formation

Every pixel in the SWIP image corresponds to one excitation laser pulse, for which, a temporal trace of the probe laser intensity will be recorded. A gating method is applied to turn the signal temporal trace to pixel intensity. Two averaging windows are used: the first window is set before the excitation pulse arriving to estimate the probe intensity baseline; the second window starts at intensity extremum right after the excitation to obtain the changed probe intensity. The pixel intensity is assigned to be the difference between the two window averages. This gating method takes advantage of the long decay of PT signal for a better SNR. Multiple window sizes have been tested and the size of 80 sampling points (~ 450 ns) is chosen to output the best SNR.

### SWIP spectroscopy

The SWIP spectra were acquired by replacing the single-color excitation laser with a tunable excitation laser (Opolette HE 355 LD, OPOTEK Inc), which has a tuning range from 410 to 2400 nm, a pulse duration of 5 ns and a repetition rate of 20 Hz. Other parts in the SWIP microscope remained unchanged. The spectral scanning was achieved by manually tuning the laser wavelength with a step size of 10 nm.

### Time domain extraction of SWIP signal from water background

The thermal property is closely related to the object size, shape, and chemical constitution. The thermal decay coefficient of SWIP signal can therefore provide extra information for differentiating small objects within bulk medium. When the particle size is smaller than the SWIP heating volume (usually satisfied for intracellular features as the axial resolution of SWIP is 3.5 μm), the heat dissipation of the small object is significantly faster than that of bulk surrounding medium. The single-pixel SWIP traces shown in Fig. 4b and 4c support this hypothesis. To reject the background from bulk medium with the thermal decay difference, every SWIP signal trace is first fitted with a two-component exponential function. Two-component exponential function is selected considering the detected SWIP signal consist of the in-focus target and out-of-focus water background contribution. A weight *w* is assigned to every pixel by soft-thresholding the decay coefficient *d* of the faster decay between the two fitted components. The final pixel intensity is calculated as the product of the assigned weight *w* and the amplitude *c* of the fitted faster decay component. The background rejection workflow is shown in **Fig. S2**.

### SRS microscope

Two synchronized femtosecond laser pulse trains with an 80 MHz repetition rate were used for SRS imaging. The wavelengths of the lasers are at 800 nm and 1040 nm to target the C-H stretch vibration. The 1040 nm laser is modulated by an acousto-optic modulator (AOM) at 2.27 MHz to separate the SRS signal from the laser repetition rate frequency. The SRS is conveyed by the modulation transfer from 1040 nm to 800 nm laser at 2.27 MHz. The two lasers are chirped to a few picoseconds with SF57 glass rods for spectral focusing. A dichroic mirror spatially combines the two lasers. The combined beams pass a pair of galvo mirrors for laser scanning, then is focused on the sample by the same objective (1.0 NA, 800 μm working distance) used for SWIP. The transmitted light is collected by a 1.4 NA oil-immersion condenser and filtered by a 980 nm short pass filter. The residue 800 nm laser is detected by a biased photodiode. The SRS signal is obtained by demodulating the signal received by a photodiode with a lock-in amplifier.

## References

[1] Kim J, Koo BK, Knoblich JA. Human organoids: model systems for human biology and medicine. Nat Rev Mol Cell Biol 2020, 21(10): 571–584. 32636524 10.1038/s41580-020-0259-3 PMC7339799

[2] Cheng JX, Xie XS. Vibrational spectroscopic imaging of living systems: An emerging platform for biology and medicine. Science 2015, 350(6264): aaa8870. 26612955 10.1126/science.aaa8870

[3] Zhang D, Li C, Zhang C, Slipchenko Mikhail N, Eakins G, Cheng J-X. Depth-resolved mid-infrared photothermal imaging of living cells and organisms with submicrometer spatial resolution. Science Advances 2016, 2(9): e1600521. 27704043 10.1126/sciadv.1600521 PMC5040478

[4] Hill AH, Manifold B, Fu D. Tissue imaging depth limit of stimulated Raman scattering microscopy. Biomed Opt Express 2020, 11(2): 762–774. 32133223 10.1364/BOE.382396 PMC7041472

[5] Esposito R, Scherillo G, Pannico M, Musto P, De Nicola S, Mensitieri G. Depth profiles in confocal optical microscopy: a simulation approach based on the second Rayleigh-Sommerfeld diffraction integral. Opt Express 2016, 24(12): 12565–12576. 27410278 10.1364/OE.24.012565

[6] Mosca S, Dey P, Salimi M, Gardner B, Palombo F, Stone N, Spatially Offset Raman Spectroscopy-How Deep? Anal Chem 2021, 93(17): 6755–6762. 33886282 10.1021/acs.analchem.1c00490

[7] Mosca S, Conti C, Stone N, Matousek P. Spatially offset Raman spectroscopy. Nature Reviews Methods Primers 2021, 1(1): 21.

[8] Schulmerich MV, Cole JH, Dooley KA, Morris MD, Kreider JM, Goldstein SA, Noninvasive Raman tomographic imaging of canine bone tissue. J Biomed Opt 2008, 13(2): 020506. 18465948 10.1117/1.2904940 PMC2658814

[9] Demers J-LH, Davis SC, Pogue BW, Morris MD. Multichannel diffuse optical Raman tomography for bone characterization in vivo: a phantom study. Biomedical Optics Express 2012, 3(9): 2299–2305. 23024921 10.1364/BOE.3.002299 PMC3447569

[10] Demers JL, Esmonde-White FW, Esmonde-White KA, Morris MD, Pogue BW. Next-generation Raman tomography instrument for non-invasive in vivo bone imaging. Biomed Opt Express 2015, 6(3): 793–806. 25798304 10.1364/BOE.6.000793 PMC4361434

[11] Wilson RH, Nadeau KP, Jaworski FB, Tromberg BJ, Durkin AJ. Review of short-wave infrared spectroscopy and imaging methods for biological tissue characterization. J Biomed Opt 2015, 20(3): 030901. 25803186 10.1117/1.JBO.20.3.030901 PMC4370890

[12] Horton NG, Wang K, Kobat D, Clark CG, Wise FW, Schaffer CB, In vivo three-photon microscopy of subcortical structures within an intact mouse brain. Nat Photonics 2013, 7(3). 10.1038/nphoton.2012.336 PMC3864872 24353743

[13] Wang HW, Chai N, Wang P, Hu S, Dou W, Umulis D, Label-free bond-selective imaging by listening to vibrationally excited molecules. Phys Rev Lett 2011, 106(23): 238106. 21770549 10.1103/PhysRevLett.106.238106 PMC3398792

[14] Wang P, Rajian JR, Cheng JX. Spectroscopic Imaging of Deep Tissue through Photoacoustic Detection of Molecular Vibration. J Phys Chem Lett 2013, 4(13): 2177–2185. 24073304 10.1021/jz400559a PMC3780401

[15] Zhao Y, Pilvar A, Tank A, Peterson H, Jiang J, Aster JC, Shortwave-infrared meso-patterned imaging enables label-free mapping of tissue water and lipid content. Nat Commun 2020, 11(1): 5355. 33097705 10.1038/s41467-020-19128-7 PMC7585425

[16] Wang LV, Yao J. A practical guide to photoacoustic tomography in the life sciences. Nat Methods 2016, 13(8): 627–638. 27467726 10.1038/nmeth.3925 PMC4980387

[17] Wang LV. Photoacoustic imaging and spectroscopy. CRC press, 2017.

[18] Hajireza P, Shi W, Bell K, Paproski RJ, Zemp RJ. Non-interferometric photoacoustic remote sensing microscopy. Light Sci Appl 2017, 6(6): e16278. 30167263 10.1038/lsa.2016.278 PMC6062239

[19] Reza PH, Bell K, Shi W, Shapiro J, Zemp RJ. Deep non-contact photoacoustic initial pressure imaging. Optica 2018, 5(7).

[20] Bell K, Mukhangaliyeva L, Khalili L, Haji Reza P. Hyperspectral absorption microscopy using photoacoustic remote sensing. Opt Express 2021, 29(15): 24338–24348. 34614681 10.1364/OE.430403

[21] Hu G, Ran Q, So BWL, Li M, Shi J, Dong X, Noncontact photoacoustic lipid imaging by remote sensing on first overtone of the C-H bond. Advanced Photonics Nexus 2023, 2(02).

[22] Cias P, Wang C, Dibble TS. Absorption Cross-Sections of the C—H Overtone of Volatile Organic Compounds: 2 Methyl-1,3-Butadiene (Isoprene), 1,3-Butadiene, and 2,3-Dimethyl-1,3-Butadiene. Applied Spectroscopy 2007, 61(2): 230–236. 17331317 10.1366/000370207779947440

[23] Bai J, Bai L, Li J, Wang Y, Xie J, Zhang D, Sensitivity Analysis of 1,3-Butadiene Monitoring Based on Space-Based Detection in the Infrared Band. Remote Sensing 2022, 14(19).

[24] Bertie JE, Lan Z. Infrared Intensities of Liquids XX: The Intensity of the OH Stretching Band of Liquid Water Revisited, and the Best Current Values of the Optical Constants of H2O(l) at 25°C between 15,000 and 1 cm – 1. Applied Spectroscopy 1996, 50(8): 1047–1057.

[25] Wang P, Wang HW, Sturek M, Cheng JX. Bond-selective imaging of deep tissue through the optical window between 1600 and 1850 nm. J Biophotonics 2012, 5(1): 25–32. 22125288 10.1002/jbio.201100102 PMC3404621

[26] Shin JY, Shaloski MA, Crim FF, Case AS. First Evidence of Vibrationally Driven Bimolecular Reactions in Solution: Reactions of Br Atoms with Dimethylsulfoxide and Methanol. J Phys Chem B 2017, 121(11): 2486–2494. 28206759 10.1021/acs.jpcb.7b00035

[27] Vardaki MZ, Kourkoumelis N. Tissue Phantoms for Biomedical Applications in Raman Spectroscopy: A Review. Biomed Eng Comput Biol 2020, 11: 1179597220948100. 32884391 10.1177/1179597220948100 PMC7440735

[28] Ishiguro T, Ohata H, Sato A, Yamawaki K, Enomoto T, Okamoto K. Tumor-derived spheroids: Relevance to cancer stem cells and clinical applications. Cancer Sci 2017, 108(3): 283–289. 28064442 10.1111/cas.13155 PMC5378268

[29] Bian X, Liu R, Meng Y, Xing D, Xu D, Lu Z. Lipid metabolism and cancer. J Exp Med 2021, 218(1). 10.1084/jem.20201606 PMC7754673 33601415

[30] Kuriu S, Kadonosono T, Kizaka-Kondoh S, Ishida T. Slicing Spheroids in Microfluidic Devices for Morphological and Immunohistochemical Analysis. Micromachines (Basel) 2020, 11(5). 10.3390/mi11050480 PMC7281316 32384758

[31] Wei M, Shi L, Shen Y, Zhao Z, Guzman A, Kaufman LJ, Volumetric chemical imaging by clearing-enhanced stimulated Raman scattering microscopy. Proc Natl Acad Sci U S A 2019, 116(14): 6608–6617. 30872474 10.1073/pnas.1813044116 PMC6452712

[32] Edwards SJ, Carannante V, Kuhnigk K, Ring H, Tararuk T, Hallbook F, High-Resolution Imaging of Tumor Spheroids and Organoids Enabled by Expansion Microscopy. Front Mol Biosci 2020, 7: 208. 33195398 10.3389/fmolb.2020.00208 PMC7543521

[33] Pampaloni F, Ansari N, Stelzer EH. High-resolution deep imaging of live cellular spheroids with light-sheet-based fluorescence microscopy. Cell Tissue Res 2013, 352(1): 161–177. 23443300 10.1007/s00441-013-1589-7

[34] Konig K, Uchugonova A, Gorjup E. Multiphoton fluorescence lifetime imaging of 3D-stem cell spheroids during differentiation. Microsc Res Tech 2011, 74(1): 9–17. 21181704 10.1002/jemt.20866

[35] Yu Y, Ramachandran PV, Wang MC. Shedding new light on lipid functions with CARS and SRS microscopy. Biochim Biophys Acta 2014, 1841(8): 1120–1129. 24576891 10.1016/j.bbalip.2014.02.003 PMC4285713

[36] Pradas I, Huynh K, Cabre R, Ayala V, Meikle PJ, Jove M, Lipidomics Reveals a Tissue-Specific Fingerprint. Front Physiol 2018, 9: 1165. 30210358 10.3389/fphys.2018.01165 PMC6121266

[37] Shi L, Fung AA, Zhou A. Advances in stimulated Raman scattering imaging for tissues and animals. Quant Imaging Med Surg 2021, 11(3): 1078–1101. 33654679 10.21037/qims-20-712 PMC7829158

[38] Taqi SA, Sami SA, Sami LB, Zaki SA. A review of artifacts in histopathology. J Oral Maxillofac Pathol 2018, 22(2): 279. 10.4103/jomfp.JOMFP_125_15 PMC6097380 30158787

[39] Urasaki Y, Zhang C, Cheng JX, Le TT. Quantitative Assessment of Liver Steatosis and Affected Pathways with Molecular Imaging and Proteomic Profiling. Sci Rep 2018, 8(1): 3606. 29483581 10.1038/s41598-018-22082-6 PMC5826939

[40] Hu S, Wang LV. Optical-resolution photoacoustic microscopy: auscultation of biological systems at the cellular level. Biophys J 2013, 105(4): 841–847. 23972836 10.1016/j.bpj.2013.07.017 PMC3752103

[41] Singh MS, Thomas A. Photoacoustic elastography imaging: a review. J Biomed Opt 2019, 24(4): 1–15. 10.1117/1.JBO.24.4.040902 PMC6990059 31041859

[42] Streich L, Boffi JC, Wang L, Alhalaseh K, Barbieri M, Rehm R, High-resolution structural and functional deep brain imaging using adaptive optics three-photon microscopy. Nat Methods 2021, 18(10): 1253–1258. 34594033 10.1038/s41592-021-01257-6 PMC8490155

[43] Tan Y, Li J, Zhao G, Huang KC, Cardenas H, Wang Y, Metabolic reprogramming from glycolysis to fatty acid uptake and beta-oxidation in platinum-resistant cancer cells. Nat Commun 2022, 13(1): 4554. 35931676 10.1038/s41467-022-32101-w PMC9356138

[44] Selmke M, Braun M, Cichos F. Photothermal Single-Particle Microscopy: Detection of a Nanolens. ACS Nano 2012, 6(3): 2741–2749. 22352758 10.1021/nn300181h

